# Pulmonary morbidity related to diaphragm surgery performed for gynecological cancers

**DOI:** 10.4274/tjod.galenos.2020.54781

**Published:** 2020-12-10

**Authors:** Yasin Durmuş, Alper Karalok, Sinem Ayşe Duru Çöteli, Nurettin Boran, Mehmet Ünsal, Gökhan Boyraz, Taner Turan

**Affiliations:** 1 University of Health Sciences Turkey, Etlik Zübeyde Hanım Women’s Health Training and Research Hospital, Clinic of Obstetrics and Gynecology, Ankara, Turkey

**Keywords:** Diaphragm, surgery, cytoreduction, ovarian cancer, morbidity

## Abstract

**Objective:**

To evaluate pulmonary morbidity related to diaphragm surgery performed for gynecological cancers and to identify the impact of transdiaphragmatic thoracotomy.

**Materials and Methods:**

We reviewed clinical and pathologic data of 232 women who had undergone diaphragm surgery as a part of cytoreductive surgery procedures performed for gynecological cancers.

**Results:**

Transdiaphragmatic thoracotomy occurred in 52 patients (22.4%). Rate of pulmonary complications among patients who had a transdiaphragmatic thoracotomy was higher compared with patients who did not have a transdiaphragmatic thoracotomy (40.4% vs 20.6%, p=0.004). Transdiaphragmatic thoracotomy [odds ratio (OR), 2.66; 95% confidence interval (CI), 1.20-5.92; p=0.016], colon resection (OR, 5.21; 95% CI, 2.34-11.63; p<0.001), ileostomy (OR, 19.61; 95% CI, 1.64-250.0; p=0.019), and any extra-pulmonary complication occurrence (OR, 2.35; 95% CI, 1.13-4.88; p=0.023) were identified as independent predictors of pulmonary morbidity. Patients with transdiaphragmatic thoracotomy developed pleural effusion, pleural effusion necessitating drainage, pneumothorax, pneumonitis, and atelectasis more frequently compared with patients who did not have transdiaphragmatic thoracotomy. Rate of admission to postoperative intensive care of patients with transdiaphragmatic thoracotomy (30.8%) was significantly higher than that of patients without transdiaphragmatic thoracotomy (12.2%) (p=0.001).

**Conclusion:**

Transdiaphragmatic thoracotomy is an independent predictor of pulmonary morbidity among patients who underwent diaphragm surgery. Avoiding accidental transdiaphragmatic thoracotomies with maximal attention and performing full-thickness resection procedures with alternative surgical techniques preventing a thoracotomy may help decrease pulmonary morbidity rates and postoperative care costs.

**PRECIS:** Transdiaphragmatic thoracotomy, colon resection, ileostomy and development of any extra-pulmonary complication were identified as independent predictors of pulmonary morbidity.

## Introduction

Studies have shown that optimal cytoreduction is the cornerstone of initial treatment for advanced epithelial ovarian cancers^(1,2)^. It is estimated that nearly 40% of patients with advanced ovarian cancer have gross disease located on the diaphragm^(3,4)^. Patients with advanced ovarian cancer and diaphragm involvement who had undergone diaphragm surgery had better survival rates than those who had not^(5)^. Thus, a significant proportion of patients with advanced ovarian cancers require diaphragm surgery to achieve optimal cytoreduction and therefore better survival rates. Complete cytoreduction is also associated with improved overall survival in advanced uterine corpus cancers, and patients with metastatic diaphragm disease require diaphragm surgery^(6,7)^.

Previous studies reported that diaphragm surgery was associated with pulmonary morbidity, and they reported higher rates of pleural effusion^(4,8,9,10,11,12)^, higher rates of pneumothorax^(8,10,11,12)^ and longer hospital stay^(4)^ after diaphragm surgery. Furthermore, several studies showed higher rates of pulmonary morbidity after diaphragm full-thickness resections compared with diaphragm peritoneal stripping^(13,14)^, but others did not show a significant difference^(9,15)^. Previous studies were not able to assess other types of pulmonary morbidity such as pneumonitis and atelectasis due to the small number of cases. Thus, pulmonary morbidity related to diaphragm surgery needs further evaluation with a larger number of cases. We performed the present study to improve knowledge about pulmonary morbidity related to diaphragm surgery, and we also assessed other potential surgical factors that may contribute to pulmonary morbidity.

## Materials and Methods

All patients who had undergone diaphragm surgery as a part of cytoreductive surgery procedures performed for advanced or recurrent ovarian and uterine corpus cancers between January 1, 2001, and June 1, 2018, were reviewed retrospectively. Data were obtained from hospital medical records. Patients who had an ablative intervention to the diaphragm were excluded from the study. We included only cases involving surgical resections of the diaphragm peritoneum with or without diaphragm muscle and overlying parietal pleura. Patients with preoperative pleural effusions and intraoperative chest tube replacements would lead to unreliable evaluation of the postoperative pulmonary complications so they were excluded.

Diaphragm debulking surgery to excise all visible metastatic tumoral deposits on the diaphragm surface was performed primarily by excision of the diaphragm peritoneum (peritoneal stripping). The extent of the diaphragm peritoneal excision was classified as “total hemi-diaphragm peritoneal stripping”, “partial hemi-diaphragm peritoneal stripping”, or “focal implant resection.” Excision of the whole hemi-diaphragm peritoneum starting from the anterior costal margin was identified as “total hemi-diaphragm peritoneal stripping”. Excision of ≤3 tumor implants ≤2 cm in diameter was identified as “focal implant resection”. All the other peritoneal excisions that were not compatible with the above definitions were classified as “partial hemi-diaphragm peritoneal stripping”.

Full mobilization of the liver was performed routinely before starting a right hemi-diaphragm total peritoneal stripping procedure. Liver mobilization was performed when needed for partial hemi-diaphragm stripping or a focal implant resection procedure depending on the location of the tumor. Tumor involvement on the left hemi-diaphragm is more easily resected without mobilizing the liver.

Diaphragm surgery may be complicated by transdiaphragmatic thoracotomy. In our study, we classified the cause of transdiaphragmatic thoracotomy as either “willful partial hemi-diaphragm full-thickness resection” or “accidental transdiaphragmatic thoracotomy”. If the surgical report indicated a histologically confirmed tumor implant invading the diaphragm muscle with or without parietal pleura, we identified the cause of the transdiaphragmatic thoracotomy as “willful partial hemi-diaphragm full-thickness resection”. If not, we identified the cause as “accidental transdiaphragmatic thoracotomy”.

Diaphragm repair was done by continuous suturing of the defect with 2/0 prolene sutures. Sutures were tied while the anesthetist was performing a forced inspiration with the help of the ventilator and the surgical team was applying a negative pressure to the thorax cavity with the help of a thin aspiration tube positioned through the remaining diaphragm defect. The second surgeon pulled out the aspiration tube as the first surgeon tied the knot. Large diaphragm defects required repair with prolene mesh.

All patients received preoperative antibiotics and anticoagulant prophylaxis. No prophylactic antibiotics were used postoperatively. Enoxaparine 4000 international units (IU) was administered 2 hours before the surgery and then repeated daily for 30 days after the surgery if the body mass index of the patient was <40 kg/m^2^; enoxaparine 6000 IU was administered if the body mass index was ≥40 kg/m^2^. All patients were assessed with a chest radiography on the first postoperative day and with physical examination daily during hospitalization. Chest radiography was repeated on the following days if any pathologic signs were detected on the physical examination. Chest computed tomography was performed when needed. Admission to the postoperative general care room or the intensive care unit was carried out according to the decision of the anesthetist. Replacement of the chest tube in the postoperative period was decided and performed by the thorax surgeon when needed. Referral to the intensive care unit during postoperative general care was decided by the senior surgeon and anesthetist when needed.

We reviewed intraoperative complications and postoperative complications that occurred within 30 days. Pulmonary morbidity was identified as the development of at least one of the following pulmonary complications: Pleural effusion, pneumothorax, pneumonitis, atelectasis, pulmonary embolism. Extra-pulmonary complications in the study group included wound infection, wound dehiscence, evisceration, intestinal anastomosis leak, ureterovaginal fistulas, vesicovaginal fistulas, gastrointestinal fistulas, ileus, acute cerebrovascular disease, chylous ascites, intraabdominal abscess, subphrenic abscess, intestinal perforation, gastric perforation, bladder perforation, pancreatic leak/fistula, appendectomy site leak, intestinal ischemia, ureter ischemia, deep venous thrombosis, arterial embolism, and myocardial infarction.

This study was approved by institutional review board (approval date and number: 21.12.2018/ 90057706-799-E.8). Signed informed consent for anonymous publication of disease related information was obtained from each patient.

### Statistical Analysis

Data were analyzed using the chi-squared test and Fisher’s Exact test for categorical variables, and the independent sample t-test was used for continuous variables. A binary logistic regression model was used for the multivariate analysis. Differences were considered significant at p<0.05. Statistical Package for Social Science (SPSS, version 17.0, SPSS, Inc., Chicago, IL, USA) was used for the statistical analyses.

## Results

We included 232 patients who had undergone diaphragm surgery. The mean age was 60.14±9.47 years (range, 36-82 years). Two hundred nine patients (90.1%) had ovarian-tubal-peritoneal cancers, 18 (7.7%) had uterine corpus cancers, 5 (2.1%) had synchronized cancers. The histopathologic subgroups are summarized in Table 1. One hundred ninety-two patients (82.8%) had undergone diaphragm surgery within the context of primary cytoreduction surgery. Forty patients (17.2%) had undergone diaphragm surgery because of recurrent cancers. Cytoreductive surgery resulted in excision of all visible tumors in 213 patients (91.8%).

One hundred eighty-three patients (78.9%) had undergone right hemi-diaphragm surgery, 2 patients (0.9%) had undergone left hemi-diaphragm surgery, and 47 patients (20.2%) had undergone bilateral hemi-diaphragm surgery. One hundred sixty-two patients (69.8%) had undergone a total hemi-diaphragm peritoneal stripping procedure, 9 patients (3.9%) had undergone a partial hemi-diaphragm peritoneal stripping procedure, 61 patients (26.3%) had undergone focal implant resection. The surgical notes reported presence of ascites in 96 patients (41.4%). Mean intraoperative blood loss was 808.3±928.1 mL. Mean operation time was 336.3±69.6 minutes. Thirty-eight patients (16.4%) needed postoperative intensive care. The median hospital stay was 9 days, and the median time from surgery to the first chemotherapy cycle was 15 days. No complications occurred in 133 patients (57.3%). Twenty-nine patients (12.5%) had pulmonary complications only, 41 patients (17.7%) had extra-pulmonary complications only, and 29 patients (12.5%) had both pulmonary and extra-pulmonary complications. Three patients died within the 30-day period after surgery, and the mortality rate was 1.3%. One patient died because of aspiration pneumonitis and 2 died as a result septic complications.

In our study, accidental transdiaphragmatic thoracotomy occurred in 20 patients (8.6%). Willful partial full-thickness excision of the hemi-diaphragm was performed in 33 patients (14.2%). One willful partial hemi-diaphragm full-thickness resection procedure was performed with a gastrointestinal staple device avoiding an entry into the pleural cavity so she was not included in the transdiaphragmatic thoracotomy group, thus remaining 52 patients (22.4%) formed the transdiaphragmatic thoracotomy group. In the study period, one large diaphragm defect required repair with prolene mesh.

We analyzed all clinicopathologic and surgical factors with regard to pulmonary morbidity. There was a higher rate of pulmonary complications when the surgical notes reported the presence of transdiaphragmatic thoracotomy. Twenty-one patients (40.4%) who had a transdiaphragmatic thoracotomy developed a pulmonary complication. Thirty-seven patients (20.6%) who did not have a transdiaphragmatic thoracotomy developed a pulmonary complication postoperatively. The difference was statistically significant (p=0.004) (Table 2). According to our data, patients who developed pulmonary complications and patients who did not develop pulmonary complications showed insignificant difference with regard to age. The mean age of patients who developed pulmonary complications was 57.4±7.9. The mean age of patients who did not develop pulmonary complications was 60.9±9.7. The difference between two groups was insignificant (p=0.108).

Patients who underwent small-bowel resection, colon resection, pelvic peritonectomy, appendectomy, tumor resection from liver Glisson’s capsule, tumor resection from the omentum minus, tumor resection from Morrison’s pouch, tumor resection from colon serosa, ileostomy, colostomy concurrently had significantly higher rates of pulmonary complication. The mean intraoperative blood loss of the patients who developed pulmonary complications was significantly higher than that of patients who did not develop any pulmonary complications (p=0.045). Patients who developed an extra-pulmonary complication had significantly higher rate of pulmonary complications compared with those who did not (p<0.001). Patients who developed an intestinal anastomosis leak had significantly higher rate of pulmonary complications (p=0.006) (Table 2).

The factors associated with pulmonary morbidity are summarized in Table 2, and we analyzed them with a binary logistic regression model. We found that transdiaphragmatic thoracotomy [odds ratio (OR), 2.66; 95% confidence interval (CI), 1.20-5.92; p=0.016], colon resection (OR, 5.21; 95% CI, 2.34-11.63; p**<**0.001), ileostomy (OR, 19.61; 95% CI, 1.64-250.0; p*=*0.019), and any extra-pulmonary complication occurrence (OR, 2.35; 95% CI, 1.13-4.88; p=0.023) were independent predictors of pulmonary morbidity (Table 3).

In our study, 48 patients (20.7%) developed pleural effusions postoperatively; 19 patients (8.2%) developed pleural effusions that necessitated drainage; 6 patients (2.6%) developed pneumothorax; 5 patients (2.1%) developed pneumonitis; 19 patients (8.2%) developed atelectasis; 5 patients (2.1%) developed pulmonary embolism. We divided the study population into 2 groups according to the absence or presence of transdiaphragmatic thoracotomy and compared the pulmonary complication subtypes separately. Patients in the transdiaphragmatic thoracotomy group developed pleural effusion, pleural effusion necessitating drainage, pneumothorax, pneumonitis, and atelectasis more frequently than patients in the no transdiaphragmatic thoracotomy group (Table 4).

Mean hospital stay, mean time interval from surgery to chemotherapy, and rate of postoperative admission to intensive care for patients with or without pulmonary complications and transdiaphragmatic thoracotomy are shown in Table 5. Patients who developed pulmonary complications had significantly longer hospital stay, longer time interval to chemotherapy, and needed postoperative intensive care more frequently. The differences in mean hospital stay and mean time interval to chemotherapy between the groups with and without transdiaphragmatic thoracotomy were insignificant. But the rate of admission to postoperative intensive care of patients with transdiaphragmatic thoracotomy was significantly higher than that of patients without transdiaphragmatic thoracotomy (Table 5).

## Discussion

Diaphragmatic surgery is performed frequently within the context of cytoreductive surgery for advanced ovarian cancers, and cytoreduction to microscopic disease improves survival. Complete cytoreduction is also associated with improved overall survival in advanced uterine corpus cancers, and patients with metastatic diaphragm disease require diaphragm surgery. When diaphragm surgery is performed, it may cause pulmonary complications and additional morbidity^(6,11)^. In this study, 25% of patients developed a pulmonary complication. Pleural effusion was the most common complication and 20.7% developed pleural effusions postoperatively; 2.6% developed pneumothorax. Other studies reported pleural effusion rates after diaphragm surgery of between 2% and 59%, and pneumothorax rates between 0% and 5%^(10,16,17)^.

Eisenhauer et al.^(9)^ analyzed pleural effusions among 52 patients who had undergone diaphragm peritonectomy or resection. They reported that ipsilateral postoperative pleural effusions occurred in 30 patients (58%). Fifteen of the 52 patients had undergone diaphragm resection or had diaphragm perforation. Eleven of 15 patients (73%) developed ipsilateral effusions. Although 73% of patients who underwent diaphragm resection and had perforations developed ipsilateral effusions, statistical analysis did not show a significantly increased rate of ipsilateral effusions after diaphragm resection or diaphragm perforation. The small number of patients in the study group and relatively high rates of pleural effusion may have led to the absence of statistical significance^(9)^. In our study, 20.7% of patients developed pleural effusions postoperatively. The rates of pleural effusion in patients without transdiaphragmatic thoracotomy and with transdiaphragmatic thoracotomy were 17.8% and 30.8%, respectively. Pleural effusion was more frequent among patients with transdiaphragmatic thoracotomy and it was statistically significant.

Soleymani Majd et al.^(15)^ compared 64 cases with diaphragmatic peritonectomy and 36 cases with pleurectomy with regard to pulmonary morbidity. The rates of pulmonary morbidity in the peritonectomy group and the pleurectomy group were 9.3% and 19%, respectively, and there was no significant difference (p=0.14). They were able to show higher rates of pulmonary morbidity after pleurectomy compared with peritonectomy, but they were unable to show a statistically significant difference due to the limited number of cases^(15)^. Ye et al.^(14)^ compared 124 patients with diaphragmatic peritonectomy and 26 cases with full-thickness diaphragmatic resection with regard to pulmonary morbidity, and they showed that patients who underwent full-thickness diaphragmatic resection developed pleural effusion significantly more frequently (25.8% versus 69.2%, p<0.001) and significantly more frequent symptomatic pleural effusion requiring drainage (8.9% versus 38.5%, p<0.001)^(14)^. Zapardiel et al.^(13)^ compared 79 patients who underwent diaphragmatic stripping and 33 patients who underwent diaphragmatic resection and showed that patients with diaphragmatic resection developed pleural effusion significantly more frequently (37.9% versus 63.6%, p=0.01).

In the current study, we were able to identify transdiaphragmatic thoracotomy as an independent predictor of pulmonary morbidity after diaphragm surgery. To our knowledge, the present study is the largest case series evaluating pulmonary morbidity related to diaphragm surgery. We were able to show that patients with transdiaphragmatic thoracotomy developed pulmonary morbidity, pleural effusions, pleural effusions necessitating drainage, pneumothorax, pneumonitis, and atelectasis significantly more frequently than patients who underwent diaphragm surgery without transdiaphragmatic thoracotomy. In the current study, all the diaphragm surgery procedures and diaphragm repairment procedures were performed by gynecological oncologists and we think that diaphragm surgery procedures can be managed by experienced gynecological oncologists. A thorax surgeon should attend the operation when a pulmonary parenchymal resection is planned. Preoperative imaging with thorax computerized tomography may help to predict cases with thorax and diaphragm involvement.

Eisenhauer et al.^(9)^ reported that ipsilateral pleural effusion was not associated with an increased length of hospital stay. Benedetti Panici et al.^(4)^ analyzed 121 patients with advanced ovarian cancer. They performed diaphragm peritonectomy in 25 patients and diaphragm resections in 43 patients and reported that diaphragmatic resection was associated with significantly longer postoperative hospital stay (median, 10.6 versus 8.1 days, p=0.005). According to our data, development of any pulmonary morbidity was associated with longer hospital stay, longer time interval to chemotherapy, and a higher rate of postoperative intensive care. Patients with transdiaphragmatic thoracotomy needed postoperative intensive care more frequently than patients without transdiaphragmatic thoracotomy. Decreasing the rate of transdiaphragmatic thoracotomy among patients undergoing diaphragm surgery may help decrease postoperative intensive care admissions and therefore reduce postoperative care costs.

Development of an extra-pulmonary complication, concurrent colon resections, and ileostomy were also independent predictors of pulmonary morbidity. Among patients with concurrent colon resections and ileostomy, prolonged intravenous support and electrolyte imbalance may have contributed to higher rates of pleural effusions and pulmonary morbidity.

### Study Limitations

The retrospective design of the study and the absence of a patient group with advanced gynecological cancer that did not undergo diaphragm surgery are limitations of the current study. The high number of patients in the study, the competence in evaluating pulmonary complications separately, and identifying independent factors associated with pulmonary morbidity by multivariate analyzes are advantages of the current study. To our knowledge, the present study is the largest case series evaluating pulmonary morbidity related to diaphragm surgery.

## Conclusion

In conclusion, diaphragm surgery helps to enhance complete cytoreduction rates and therefore improves survival in advanced epithelial ovarian cancers and uterine corpus cancers. İt is associated with higher rates of pulmonary complications. Transdiaphragmatic thoracotomy is an independent predictor of pulmonary morbidity among patients who undergo diaphragm surgery. Avoiding accidental transdiaphragmatic thoracotomies with maximal attention may help decrease pulmonary morbidity rates and postoperative care costs. Potential benefits of alternative surgical techniques (e.g using staple devices) preventing entry into the thorax cavity while performing full-thickness diaphragm resections should further be investigated.

## Figures and Tables

**Table 1:**
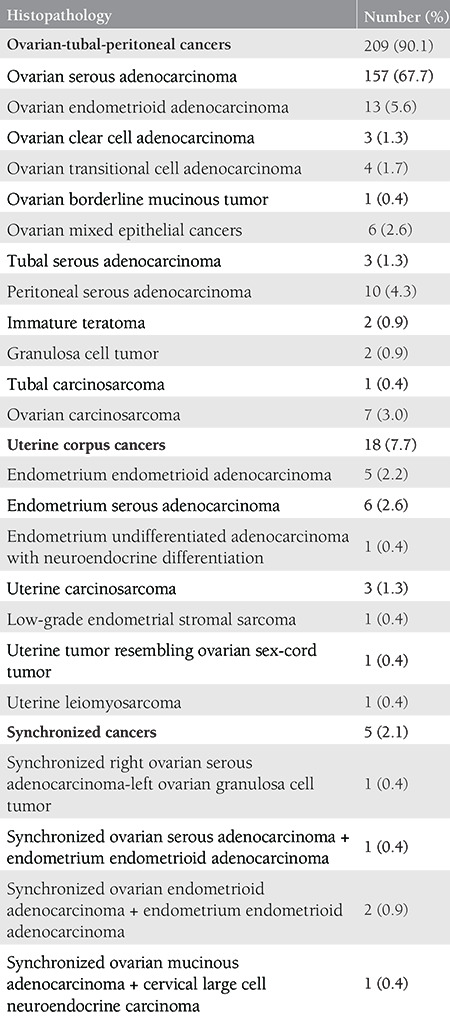
Histopathologic characteristics of the study group

**Table 2:**
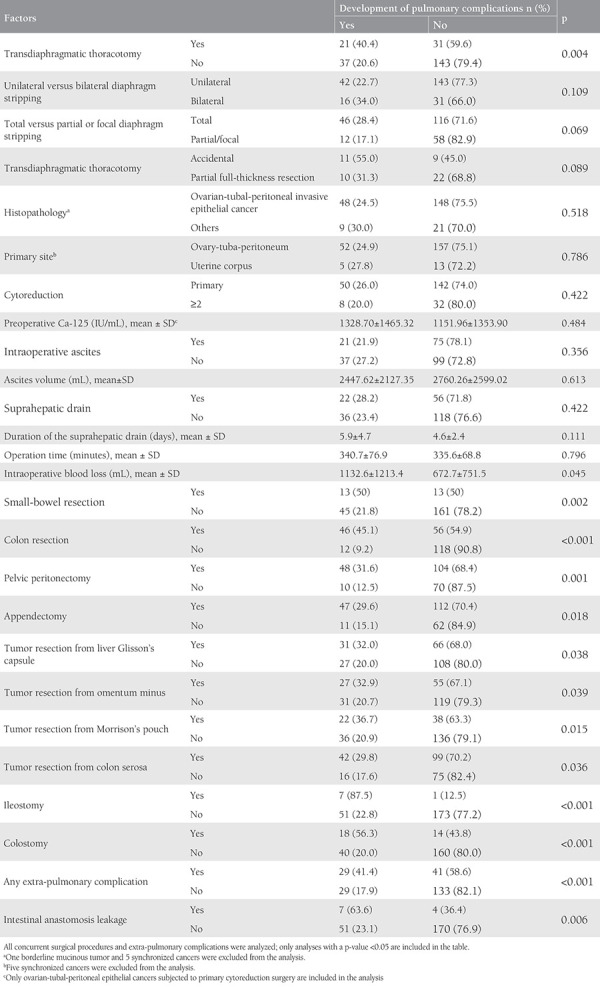
Factors associated with pulmonary morbidity

**Table 3:**
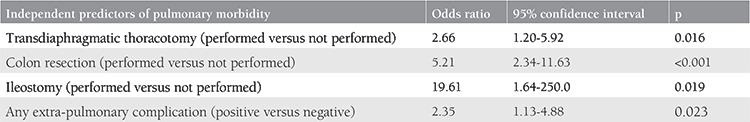
Independent predictors of pulmonary morbidity

**Table 4:**
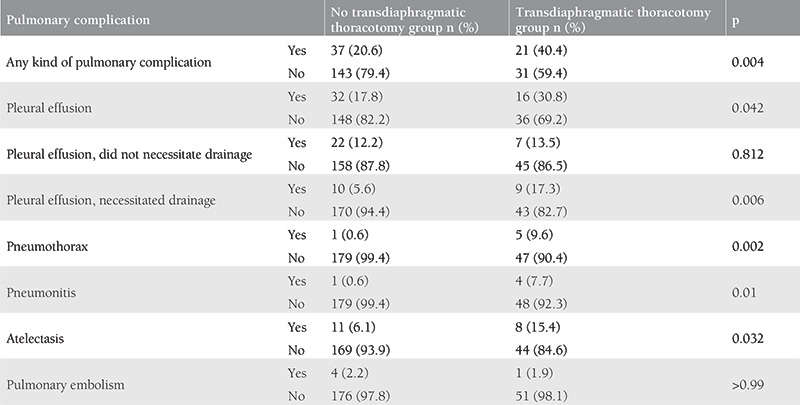
Comparison of pulmonary complications in patients with and without transdiaphragmatic thoracotomy

**Table 5:**
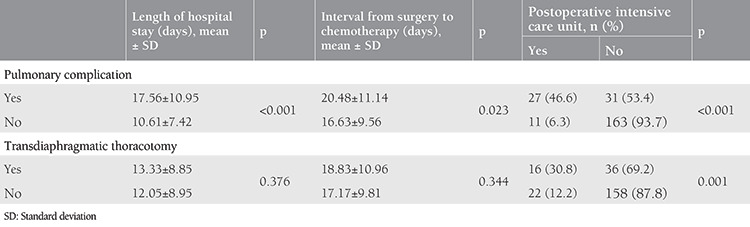
Mean length of hospital stay, mean time from surgery to chemotherapy, and rate of postoperative intensive care

## References

[1] Bristow RE, Tomacruz RS, Armstrong DK, Trimble EL, Montz FJ (2002). Survival effect of maximal cytoreductive surgery for advanced ovarian carcinoma during the platinum era: a meta-analysis. J Clin Oncol.

[2] Chi DS, Eisenhauer EL, Lang J, Huh J, Haddad L, Abu-Rustum NR, et al (2006). What is the optimal goal of primary cytoreductive surgery for bulky stage IIIC epithelial ovarian carcinoma (EOC)?. Gynecol Oncol.

[3] Papadia A, Morotti M (2013). Diaphragmatic surgery during cytoreduction for primary or recurrent epithelial ovarian cancer: a review of the literature. Arch Gynecol Obstet.

[4] Benedetti Panici P, Di Donato V, Fischetti M, Casorelli A, Perniola G, Musella A, et al (2015). Predictors of postoperative morbidity after cytoreduction for advanced ovarian cancer: analysis and management of complications in upper abdominal surgery. Gynecol Oncol.

[5] Aletti GD, Dowdy SC, Podratz KC, Cliby WA (2006). Surgical treatment of diaphragm disease correlates with improved survival in optimally debulked advanced stage ovarian cancer. Gynecol Oncol.

[6] Alagkiozidis I, Grossman A, Tang NZ, Weedon J, Mize B, Salame G, et al (2015). Survival impact of cytoreduction to microscopic disease for advanced stage cancer of the uterine corpus: a retrospective cohort study. Int J Surg.

[7] Solmaz U, Mat E, Dereli ML, Turan V, Ekin A, Tosun G, et al (2016). Stage- III and -IV endometrial cancer: a single oncology centre review of 104 cases. J Obstet Gynaecol.

[8] Fanfani F, Fagotti A, Gallotta V, Ercoli A, Pacelli F, Costantini B, et al (2010). Upper abdominal surgery in advanced and recurrent ovarian cancer: role of diaphragmatic surgery. Gynecol Oncol.

[9] Eisenhauer EL, D’Angelica MI, Abu-Rustum NR, Sonoda Y, Jarnagin WR, Barakat RR, et al (2006). Incidence and management of pleural effusions after diaphragm peritonectomy or resection for advanced Mullerian cancer. Gynecol Oncol.

[10] Devolder K, Amant F, Neven P, van Gorp T, Leunen K, Vergote I (2008). Role of diaphragmatic surgery in 69 patients with ovarian carcinoma. Int J Gynecol Cancer.

[11] Chereau E, Rouzier R, Gouy S, Ferron G, Narducci F, Bergzoll C, et al (2011). Morbidity of diaphragmatic surgery for advanced ovarian cancer: retrospective study of 148 cases. Eur J Surg Oncol.

[12] Bashir S, Gerardi MA, Giuntoli RL, Montes TPD, Bristow RE (2010). Surgical technique of diaphragm full-thickness resection and trans-diaphragmatic decompression of pneumothorax during cytoreductive surgery for ovarian cancer. Gynecol Oncol.

[13] Zapardiel I, Peiretti M, Zanagnolo V, Biffi R, Bocciolone L, Landoni F, et al (2011). Diaphragmatic surgery during primary cytoreduction for advanced ovarian cancer: peritoneal stripping versus diaphragmatic resection. Int J Gynecol Cancer.

[14] Ye S, He T, Liang S, Chen X, Wu X, Yang H, et al (2017). Diaphragmatic surgery and related complications in primary cytoreduction for advanced ovarian, tubal, and peritoneal carcinoma. BMC Cancer.

[15] Soleymani Majd H, Ferrari F, Manek S, Gubbala K, Campanile RG, Hardern K, et al (2016). Diaphragmatic peritonectomy vs. full thickness resection with pleurectomy during Visceral-Peritoneal Debulking (VPD) in 100 consecutive patients with stage IIIC-IV ovarian cancer: a surgical-histological analysis. Gynecol Oncol.

[16] Gouy S, Chereau E, Custodio AS, Uzan C, Pautier P, Haie-Meder C, et al (2010). Surgical procedures and morbidities of diaphragmatic surgery in patients undergoing initial or interval debulking surgery for advanced-stage ovarian cancer. J Am Coll Surg.

[17] Dowdy SC, Loewen RT, Aletti G, Feitoza SS, Cliby W (2008). Assessment of outcomes and morbidity following diaphragmatic peritonectomy for women with ovarian carcinoma. Gynecol Oncol.

